# Drug-Resistance Associated Mutations in Polymerase (P) Gene of Hepatitis B Virus Isolated From Malaysian HBV Carriers

**DOI:** 10.5812/hepatmon.13173

**Published:** 2014-01-05

**Authors:** Jeyanthi Suppiah, Rozainanee Mohd Zain, Salbiah Haji Nawi, Norazlah Bahari, Zainah Saat

**Affiliations:** 1 Virology Unit, Institute for Medical Research, Kuala Lumpur, Malaysia; 2 Microbiology Unit, Hospital Kuala Lumpur, Kuala Lumpur, Malaysia; 3 Pathology Unit, Selayang Hospital, Selangor, Malaysia

**Keywords:** Gene Products, pol, Drug Resistance, Hepatitis B, Mutation, Lamivudine, Genotype

## Abstract

**Background:**

Mutations in the polymerase (P) gene of hepatitis B virus are often associated with drug resistance. The pattern of mutations varies geographically, thus giving rise to genotypes diversity.

**Objectives:**

This study was carried out to detect mutations in P gene of hepatitis B virus isolated from Malaysian HBV carriers.

**Materials and Methods:**

A total of 58 sera samples were analyzed by PCR and sequencing, of which the P gene of isolated HBV was successfully amplified and sequenced from 40 samples.

**Results:**

Genotyping of these samples revealed that the predominant genotype was genotype C (22/40, 55.0%), followed by genotype B (17/40, 42.5%), and only 1 sample showed genotype D (2.5%). A number of significant drug resistant mutations were found in five patients including S202I, N236T, M250L, L180M/V, M204I, A181T, T184G, M250V, and V173L. Of these, L180M/V and M204I were most frequently detected (80%) and associated with lamivudine in combination with emtricitabine and telbivudine drug resistance. Association with age, sex, and clinical symptoms revealed that these patients were all male, mid to elderly age and almost all hadcirrhotic liver disease.

**Conclusions:**

Detection and surveillance of the significant sites of mutations in HBV is crucial for clinicians to decide on the choice of antiviral treatment and further management of hepatitis B carriers.

## 1. Background

Infection with Hepatitis B virus (HBV) is a major global health problem affecting over 350 million people in the world (1). HBV is known to cause genomic integration in liver tissue resulting in chromosomal deletions and rearrangements ensuing in metaplasia. Complications arising from chronic infection include liver cirrhosis and hepatocellular carcinoma (HCC). In Malaysia, HBV is known as main etiological agent causing HCC, while HCC is the 7th leading cause of death due to cancers. HBV is transmitted by perinatal, percutaneous, and sexual exposure, as well as by close person-to-person contact presumably by open cuts and sores, especially among children in hyper endemic areas (1).

Due to high mutational rates in the HBV-DNA, the virus could be classified into several genotypes with a standard cut-off of 8% genomic divergence (2). The most common mutation involved substitution of methionine for valine or isoleucine rtM204V/I (3). Very little studies were carried out to detect mutations in HBV isolated from Malaysian population. Study on the mutation in the core and precore promoter region of HBV in Malaysian carriers has been demonstrated (4). Apart from that, mutation in the immuno-dominant region of pre S and S gene in HBV from infected Malaysian patients has also been studied (5).

## 2. Objectives

However, no findings were reported on the mutations in polymerase (P) gene of HBV in Malaysia. Therefore, this study focused on sequencing and mutation analysis of P gene of HBV virus in infected Malaysian patients.

## 3. Materials and Methods

### 3.1. Ethics Statement

This study was ethically approved by Ethics and Medical Research Committee, Ministry of Health, Malaysia (Reference number: NMRR-12-311-11789). The ethics committee deemed that patient consent was not required as the samples used were retrospective and confirmed as Hepatitis B carriers positive by Hospital Selayang, Kuala Lumpur, Malaysia.

### 3.2. Samples

A total number of 58 blood serum samples of confirmed Hepatitis B carriers receiving antiviral treatment were obtained in a volume of 2 mL from Pathology Unit, Hospital Selayang, Kuala Lumpur, Malaysia. Hepatitis B carriers were defined as persons positive for Hepatitis B surface antigen (HBs Ag) for more than six months. The serum samples obtained were retrospective samples collected for a period of two years (2011-2012). Patients were chosen randomly regardless of age, race, sex, and symptoms. 

### 3.3. Viral-DNA Isolation

HBV DNA isolation was performed using High Pure Viral Nucleic Acid Extraction Kit (Roche, USA) according to the manufacturer’s instructions. The isolation procedure was based on spin-column method. The final elution volume of 50 µL containing viral RNA from each sample was stored at -20ºC for long-term usage.

### 3.4. Polymerase (P) Gene PCR Amplification

Approximately 2.5 kb length of polymerase (P) gene of HBV was amplified using 10 sets of published oligonucleotides (6). All oligonucleotides used in this study are listed in Table 1 and Table 2. A fragment of 3.2 kb was amplified in the first round PCR using sense HB8F and antisense HB6R. A second round of PCR using 9 sets of oligonucleotides (HB1F-HB1R, HB2F-HB2R, HB3F-HB3R, HB4F-HB4R, HB5F-HB5R, HB9F-HB9R, HB10F-HB10R, HB11F-HB11R, HB12F-HB12R) were performed on the 3.2 kb fragment to produce 9 overlapping fragments that contributed to full length P gene sequence when aligned. All amplification reactions were carried out in a 96-well Thermal Cycler (Bio Rad, USA). The first round of PCR was undertaken for 35 cycles (94 °C for 1 min, 55 °C for 1 min, and 72 °C for 1.5 min) followed by an extension reaction at 72 °C for 5 min. The second round PCR was performed for 30 cycles (94 °C for 1 min, 55 °C for 1 min, and 72 °C for 1 min) followed by extension at 72 °C for 5 min. First round PCR reaction was composed of 12.5 uLof 2x MiFi Mix, 1.0 uLof each oligonucleotides (10 uM), 5.5 uL sterile dH2O, and 5 uL of extracted HBV DNA. The second round PCR reaction was composed of the same reagent concentrations for each of 9 sets of oligonucleotides, except that only 2 uL of the first round PCR product was used as template. A synthetic Hepatitis B virus isolate M1 (Gen Bank Accession number: GQ924603) was used as positive control in the initial run to test the primers. A negative control was also included in each run of PCR.

**Table 1.** Oligonucleotides Used in First Round PCR

Oligonucleotide Identity	Sequence, (5’-3’)	Reference
**HB8F**	TTCACCTCTGCCTAATCATC	Sugauchi et al. (2001) (6)
**HB6R**	AACAGACCAATTTATGCCTA	Sugauchi et al. (2001) (6)

**Table 2.** Oligonucleotides Used in Second Round PCR

Oligonucleotide Identity	Sequence, (5’-3’)	Reference
**HB1F**	AAGCTCTGCTAGATCCCAGAGT	Sugauchi et al. (2001) (7)
**HB1R**	GAAACATAGAGGTGCCTTGAGCAG	Sugauchi et al. (2001) (7)
**HB2F**	TGCTGCTATGCCTCATCTTC	Sugauchi et al. (2001) (7)
**HB2R**	CATACTTTCCAATCAATAGG	Sugauchi et al. (2001) (7)
**HB3F**	GCCAAGTCTGTACAACATCTTGAG	Sugauchi et al. (2001) (7)
**HB3R**	AGTTGGCGAGAAAGTGAAAGCCTG	Sugauchi et al. (2001) (7)
**HB4F**	CCTATTGATTGGAAAGTATGTCA	Sugauchi et al. (2001) (7)
**HB4R**	CGGGACGTAGACAAAGGACGT	Sugauchi et al. (2001) (7)
**HB5F**	CTCTGCCGATCCATACTGCGGAA	Sugauchi et al. (2001) (7)
**HB5R**	TTAACCTAATCTCCTCCCCCA	Sugauchi et al. (2001) (7)
**HB9F**	TCAGGCAACTATTGTGGTTTCA	Sugauchi et al. (2001) (7)
**HB9R**	GGATAGAACCTAGCAGGCAT	Sugauchi et al. (2001) (7)
**HB10F**	CGCAGAAGATCTCAATCTCGG	Sugauchi et al. (2001) (7)
**HB10R**	GGGTTGAAGTCCCAATCTGGATT	Sugauchi et al. (2001) (7)
**HB11F**	GGGTCACCATATTCTTGGGAA	Sugauchi et al. (2001) (7)
**HB11R**	GAACTGGAGCCACCAGCAGG	Sugauchi et al. (2001) (7)
**HB12F**	GTGGAGCCCTCAGGCTCAGG	Sugauchi et al. (2001) (7)
**HB12R**	CGAGTCTAGACTCTGTGGTA	Sugauchi et al. (2001) (7)

### 3.5. Post PCR Purification and Sequencing

A 10 uL aliquot of each PCR reaction from the second round PCR was analyzed on 2 % agarose by gel electrophoresis and viewed under UV illumination. The agarose was pre-stained with Red Safe Dye (Intron Biotech, Korea) as an alternative to Ethidium Bromide. The corresponding amplicons were extracted from the agarose gel and purified using Gel Extraction Kit (Qiagen, USA) according to the manufacturer’s instruction. Final elution contained 35 uL of purified PCR amplicons from which 5 uL was reanalyzed on 2 % agarose gel to confirm that the purification step was performed precisely. All purified PCR amplicons and corresponding sense and antisense oligonucleotides were sent to NHK Bioscience for sequencing.

### 3.6. Genotyping Malaysian Hepatitis B Carriers

Overlapping sense and antisense sequences retrieved because of sequencing were aligned to produce a full length P gene sequence of HBV using CLUSTALW software (http://www.ebi.ac.uk/Tools/msa/clustalw2/). The reference sequence used in the alignment was Hepatitis B virus isolate M1 (Gen Bank Accession number: GQ924603). Subsequently, derived P gene sequences were used to determine genotype of Malaysian Hepatitis B carriers using Genotyper bioinformatics tool in HEPSEQ, International Repository for Hepatitis B Virus Strain Data

(http://www.hpa-bioinformatics.org.uk/HepSEQ-Research/Public/Tool/genotype_tool.php).

### 3.7. Analyzing Important Mutations in P Gene

The P gene full length sequences were analyzed for presence of significant mutations using HEPSEQ polymerase annotator tool. This bioinformatics tool provided the information for mutation and it’s indication in a given Hepatitis B sequence. 

## 4. Results

### 4.1. PCR Amplification of P Gene 

**Figure 1.**
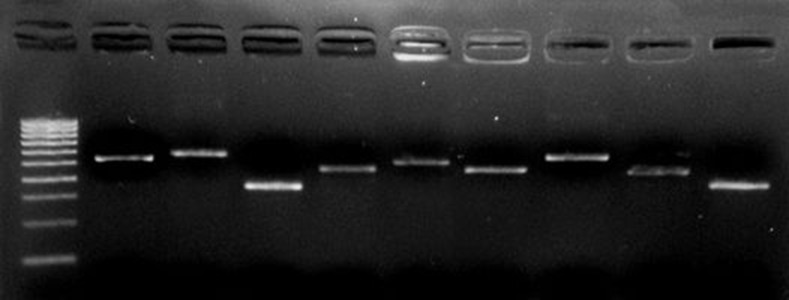
Amplification of P Gene Full Sequence by Fragments in Hepatitis B Samples of a Patient LANE1, 100 bp ladder; LANE 2, HB1(540 bp); LANE 3, HB2(576 bp); LANE 4, HB3(348bp); LANE 5, HB4(465 bp); LANE 6, HB5(506 bp); LANE 7, HB9(465bp); LANE 8, HB10(571 bp); LANE 9, HB11(462bp); LANE 10, HB12(382bp); Amplicons shown were related to purified PCR products.

P gene amplification was observed in all 40/58 Hepatitis B serum samples. The remaining 18 sera showed negative amplification. The P region was amplified fragment by fragment with accurate amplicon sizes as shown in Figure 1. 

### 4.2. P Gene Sequences of Hepatitis B

Alignment of forward and reverse sequences of each fragment of P gene against the reference strain produced a full length of 2532 bp of P gene. The sequences were deposited in Gen Bank (Accession numbers: KF053159- KF053194).

### 4.3. Malaysian Hepatitis B Genotype Analysis

Based on P gene sequences, genotyping results revealed that, of 40 Hepatitis B patients, 22 belonged to genotype C (55.0%), 17 were genotype B (42.5%), and 1 was genotype D (2.5%).

### 4.4. Case Report of Mutations in P Gene of Hepatitis B

Analysis of P gene sequence revealed that genome of five patients contained mutations that caused drug resistance. The mutations found are as listed in Table 3 and the characteristics of these patients are summarized in Table 4. Based on genome analysis, Patient 155693 possessed mutation A181T, which was responsible to cause resistance to lamivudine. The disease of this patient, a 64-year-old man, was diagnosed as chronic hepatitis B and was on lamivudine treatment from May 2006-February 2007, then restarted from May 2007 onwards. In 2008, adefovir was added as an antiviral therapy. The patient was followed up until November 2012 and HBV DNA was still detected.

**Table 3.** List of P Gene Mutations Found in Patients in This Study and Association with Drug Resistance

P Gene Mutations	Patient Identification	Association With Drug Resistance
**S202I**	830757	Entecavir resistant
**N236T**	830757	Adefovir resistant
**M250L**	830757	Entecavir resistant
**L180M/ L180V**	247131, 630304, 839920	Lamivudine, Emtricitabine and Famciclovir resistant
**M204I**	247131, 630304, 839920	Lamivudine, Emtricitabine and Telbivudine resistant
**A181T**	155593	Lamivudine and Adefovir resistant
**T184G**	247131	Entecavir resistant
**M250V**	247131	Entecavir Resistant
**V173L**	839920	Lamivudine, Emtricitabine and Telbivudine resistant

**Table 4.** Characteristics Summary of the Patients Who Carry HBV, P Gene Mutation

Patient ID	Sex	Age	History of Treatment	HBV Viral Load	Clinical Diagnosis
**155693**	Male	64	-On Lamivudine since May 2006 –Jan 2007, No treatment Feb 2007-May 2007, Adefovir added on Sept 2008 – Nov 2012, Non-compliant with meds, No follow up since Nov 2012	Before treatment: 5.4 × 107 iu/mL, Last reading in Apr 2012: < 20 iu/mL	Chronic Hepatitis B, U/S abdomen showed fatty liver with multiple cysts and cholelithiasis, No cirrhosis
**247131**	Male	48	-On tenofovir since Dec 2011,Also treated with Ciprofloxacin IV on discharge for Spontaneous bacterial peritonitis, No history of treatment failure, No follow up after discharge (Jan 2012)	Not available	-Decompensated liver cirrhosis, U/S abdomen showed splenomegaly, gross ascites, and pleural effusion
**830757**	Male	28	- Referred from another hospital for further management of Hep B, -No information available on the antiviral treatment, Ptimproved well, no HBV DNA detected , biochemical parameters showed improving trend	Not available	-Possible drug induced hepatitis, No liver cirrhosis, Present with jaundice
**630304**	Male	46	-Initially on Lamivudine since June 2009, later switched to tenofovir from July 2011 till May 2012 due to failure of viral suppression	Before treatment: 7.6 × 107 iu/mL, Last reading before switching to tenofovir:1.59 × 107 iu/mL, Three months after receiving tenofovir: 1.0 × 103 iu/mL, One year after receiving tenofovir: < 6 iu/ml	Liver cirrhosis secondary to chronic Hepatitis B, Pt keeping well and no signs of liver decompensation after receiving tenofovir
**839920**	Male	76	-Started with tenofovir since Nov 2011, stopped after 1 month, -Readmitted in May 2012, Admitted to palliative care unit in Feb 2013	Before treatment: 2.86 × 105 iu/mL, After treatment: Not available	Initial OGDS showed multiple hematin-based ulcers in stomach and duodenum, U/S revealed liver cirrhosis with nodular outline, Diagnosed as advanced hepatitis B Hepatocellular Carcinoma complicated with liver cirrhosis, Patient passed away in Feb 2013

Patient 247131 was found to have mutations L180M, M204I, T184G, and M250V, which were associated with resistance to lamivudine, entecavir, emitricitabine, famciclovir, and telbivudine. The disease of a 48-year-old male was diagnosed as decompensated liver cirrhosis. The patient was treated with tenofovir since Dec 2011 and no history of treatment failure found. The patient was not followed up thereafter. 

Patient 830757 also had multiple mutations in HBV P gene, which were S202I, N236T, and M250L. These mutations were known to cause resistance towards entecavir and adefovir. This patient, a 26-year-old male, initially diagnosed with possible drug induced hepatitis in 2011 by Sultanah Aminah Hospital, was sent to Selayang Hospital for further management of Hepatitis B/C. When patient was readmitted in 2012, no HBV DNA was detected, therefore discharged within three days of admission. No information was available on the antiviral treatment given to this patient.

Analysis of the HBV P gene sequence of patient 630304 showed presence of two significant mutations which were L180M and M204I, indicating possible multi-drug resistance to lamivudine, emtricitabine, famciclovir, and telbivudine. The patient, a 46-year-old man, was presented with liver cirrhosis secondary to chronic HBeAg positive. The patient initially started on lamivudine for a period of two years, then switched to tenofovir mid of 2011 onwards due to failure of viral suppression. It was also stated that no resistant test was available at that time. As of May 2012, HBV DNA was still detected, however the viral load had remarkably descended from approximately 15, 900, 000 IU/mL (just before switching to tenofovir) to < 6 IU/mL. The patient was reported thereafter to have no more symptoms or signs of liver decompensation.

Based on sequence analysis, patient 839920, a 76-year-old male, showed the presence of L180V, M204I, and V173L mutations in P gene sequence of HBV DNA. These mutations were associated with resistance to lamivudine, emtricitabine, telbivudine, and entecavir.Full sequence amplification failed for this particular patient, therefore only partial sequence was retrieved. Clinical history of the patient revealed liver cirrhosis and ascites. The patient was on tenofovir treatment since 2011 but was non-compliant. Patient’s health condition was deteriorating and he passed away in February 2013 due to advanced hepatocellular carcinoma.

## 5. Discussion

The HBV virus is currently genotyped into ten (A-J) genotypes (7). The prevalence of HBV genotypes varies depending on the geographical distribution (4). Genotype A is prevalent in the United States, Northern Europe, Central Africa, and Latin America. Genotype B and C were dominant in the Asian Region, while Genotype D had a worldwide distribution but most prevalent in the Mediterranean and Western Asian countries. The distribution of genotype E and F was reported in West Africa and United States respectively, whereas genotype G was only detected in France and United States. The presence of genotype H was reported in Central America (8). In 2008 a new variant of genotype was added namely genotype I (9), while in 2009, genotype J was isolated from a Japanese patient (10). 

In our study, it has been found that genotype C (55.0%) was dominant but only with a slightly higher prevalence than genotype B (42.5%). This finding differs from another study (11) that reported 53.4% of the Malaysian HBV positive was found to be of genotype B, followed by 13.6% of genotype C and 1.1% of genotype D and E. In another study conducted in Malaysia population, equal distribution of genotype B and C was found, however the sample size was too small (5). Our study result was comparable with many previous reports on the geographical distribution of HBV genotypes, whereby both genotypes B and C were most prevalent in Asia (12, 13). Among our study population, only one patient was found with genotype D. The HBV isolated from this patient contained a 31-nucleotide deletion at the location which was also the beginning of PreS1open reading frame. This deletion has been reported exclusively for in Genotype D of Hepatitis B and non-human primate isolates (14). Based on ethnicity, HBV genotype C was equally distributed among Malay and Chinese patients. However, genotype B was seen more frequently (64.7%) in Chinese people. Similar finding was reported with frequency of 80% of genotype B among Malaysian Chinese (15).

The HBV genomic variations in P gene region have clinical importance. The mutations in this region have been reported to have association with drug resistance in patients. For antiviral therapy, five nucleoside/nucleotide analogues (lamivudine, adefovir, entecavir, telbivudine, and tenofovir) are approved at the present by European Association for the Study of Liver. Lamivudine resistance mutants were reported to harbor M240V/I in the YMDD motif of the polymerase gene (16). Adefovir resistance was caused by N236T and /or A181V amino acid substitution (17), whereas entecavir resistance resulted from HBV reverse transcriptase substitutions at positions T184, S202, or M250L which emerge in the presence of lamivudine resistance substitutions M204I/V and L180M (18). Resistance to telbivudine has been associated with M204I mutation (19). Emtricitabine resistance was often accompanied with lamuvudine resistance because of L180M, V173L, and M204I mutations (20).

The results of this study have demonstrated drug resistant HBV mutations in five patients. All five patients were male with the age ranging from 26-76 years. Based on the genome analysis, HBV isolated from four out of five patients (80%) contained mutations associated with lamivudine resistance in combination with either of emtricitabine, telbivudine, or adefovir. Another study has also reported high frequency of lamivudine resistance in up to 70% of HBV patients who were treated 5 years with lamivudine, 29% after 5 years with adefovir, 20% after 2 years with telbivudine, and 1% after 5 years with entecavir (21). The mutations found in the four patients were L180M/V, M204I, A181T, and V173L.Among these, L180M/V and M204I were frequently observed. Some study reported that in most cases, the M204V/I mutation was not present alone but linked with a leucine to methionine exchange at position 180 (L180M) (22). Similarly, three out of five patients contained both M204I and L180M/V. Mutations associated with entecavir and adefovir resistance were the next frequently observed in two patients each (40%). 

It was observed that the four HBV isolates (247131, 630304, 839920, and 155593) with lamivudine resistant mutations were all from patients who were on lamivudine treatment at some points. Interestingly, two patients have completely recovered after switching of antiviral therapy from lamivudine to tenofovir. This indicates the efficacy of tenofovir as treatment for HBV patients. This finding is supported by Fung et al. (2012) (23) stating that tenofovir has shown high barrier to resistance, produced high rates of viral suppression, and showed no evidence of resistance through six years for previously untreated chronic hepatitis B patients. Similar finding was also demonstrated whereby regression of cirrhosis during treatment was observed with tenofovir in chronic hepatitis B patients in a five-year follow-up (24). The remaining two patients did not recover, as one patient was still on lamivudine treatment and the other died recently. The dead patient was reported to have been on tenofovir treatment since 2011, however was non-compliant, which could be the possible cause of treatment failure. Two patients had HBV mutant associated with adefovir resistance (830757 and 155593), in one patient of which adefovir treatment as second-line treatment with lamivudine was confirmed. Genomic analysis revealed that HBV from this patient also had lamivudine resistant mutation. This finding supports earlier studies (25) that demonstrated adefovir resistance occurred more frequently in second-line treatment of lamivudine-resistant patients than in naive patients. Therefore administration of adefovir in patients with history of lamivudine resistance should be abandoned. The other patient’s treatment history was unclear, but there was a possibility that this patient has also received adefovir treatment.

Analysis on symptoms of five patients with HBV P gene mutants revealed thatthe disease of three patients were diagnosed as liver cirrhosis and the remaining two as chronic hepatitis B and drug induced hepatitis, respectively. All three patients with liver cirrhosis contained HBV mutant L180M in combination with M204I. However, to conclude if there is any association of hepatitis B disease symptoms with the presence of drug resistant mutants, larger sample sizes need to be used. Larger sample size would be an advantage for this study. However, the findings in this study has potential clinical impact, as none of the drug resistance mutations were reported in Malaysian patients neither have they been published.

In conclusion, genomic analysis of HBV P gene isolated from HBV carriers could reveal abundant information including mainly the presence of HBV drug resistant mutants which could lead to prediction of effectiveness of antiviral therapy as well as severity of the disease. Therefore, it is of importance to evaluate antiviral therapy by surveillance of the significant sites of mutations. Early detection of HBV drug resistance is crucial for clinicians to decide on the choice of antiviral treatment and further management of Hepatitis B carriers.
